# Home the Place to Play

**Published:** 1890-10

**Authors:** 


### HOME THE PLACE TO PLAY.

The downfall of many a man or woman can be traced to a beginning with depraved associates. Yet how many parents in villages or cities know where their boy is from 7 to 10 P. M. every night in the week? The strongest boy, morally, cannot withstand the pressure brought to bear upon him night after night by vile associates, and will inevitably imbibe many wretched, vicious ideas. The formative period of child life is between the ages of five and fourteen years—the very age when a boy is of least use at home, when he is most in the way, when he is so full of animal spirits that it is impossible to repress him. Don't try to do it. Let the surplus energy escape by activity in the home. Is not the temporal and eternal welfare of your boy worth more than all the furniture? We have no room in our house too good for the children. Make home the happiest place on earth for your boy—books, music, games; let him have his associates frequently; let them play games (under your own watchful eye) even if they are a little rough; better that than drive your boy into the street. Play with them, it will not hurt your dignity; you are, in great measure, responsible for the future welfare of your child.—*Farm and Home.*—*Eng.*

### THE DREAD OF DEATH.

Sir Lyon Playfair, in a letter to Junius Henri Browne, says:

I have known three friends who were partially devoured by wild beasts under apparently hopeless circumstances of escape. The first was Livingstone, the great African traveler, who was knocked on his back by a lion, which began to munch his arm. He assured me that he felt no pain or fear, and that his only feeling was one of intense curiosity as to which part of his body the lion would take next. The next was Rustem Pasha, now Turkish ambassador in London. A bear attacked him and tore off part of his hand and part of his arm and shoulder. He also assured me that he had neither pain nor fear, but that he felt excessively angry because the bear grunted with so much satisfaction in munching him.

The third case is that of Sir Edward Bradford, an Indian officer, now occupying a high position in the Indian service. He was seized in a solitary place by a tiger, which held him firmly behind the shoulders with one paw and then deliberately devoured the whole of his arm, beginning at the end and finishing at the shoulder. He was positive that he had no sensation of fear, and thinks that he felt a little pain when the fangs went through his hand, but is certain that he felt none during the munching of his arm.

